# Development of Chlorantraniliprole and Lambda Cyhalothrin Double-Loaded Nano-Microcapsules for Synergistical Pest Control

**DOI:** 10.3390/nano11102730

**Published:** 2021-10-15

**Authors:** Boyuan Feng, Heng Zhi, Hongyan Chen, Bo Cui, Xiang Zhao, Changjiao Sun, Yan Wang, Haixin Cui, Zhanghua Zeng

**Affiliations:** Institute of Environment and Sustainable Development in Agriculture, Chinese Academy of Agricultural Sciences, Beijing 100081, China; fengboyuan@caas.cn (B.F.); 82101171041@caas.cn (H.Z.); chenhongyan0103@126.com (H.C.); cuibo@caas.cn (B.C.); zhaoxiang@caas.cn (X.Z.); sunchangjiao@caas.cn (C.S.); wangyan03@caas.cn (Y.W.); cuihaixin@caas.cn (H.C.)

**Keywords:** microcapsules, double-loaded, synergistic effect, pest control

## Abstract

Nanotechnology could greatly improve global agricultural food production. Chlorantraniliprole and lambda cyhalothrin double-loaded nano-microcapsules were fabricated to enhance the control of pests by pesticides and improve the pesticide utilization efficiency. The nano-microcapsules were synthesized using a method involving the solid in oil in water encapsulation technique and solvent evaporation. The nano-microcapsules slowly and simultaneously released lambda cyhalothrin and chlorantraniliprole. The cumulative lambda cyhalothrin and chlorantraniliprole release rates at 40 h were 80% and 70%, respectively. Indoor *Spodoptera frugiperda* control tests indicated that the double-loaded nano-microcapsules were more toxic than lambda cyhalothrin water-dispersible granules, chlorantraniliprole water-dispersible granules, and a mixture of lambda cyhalothrin water-dispersible granules and chlorantraniliprole water-dispersible granules, indicating that the pesticides in the nano-microcapsules synergistically controlled *Spodoptera frugiperda*. The results indicated that pesticide nano-microcapsules with synergistic effects can be developed that can improve the effective pesticide utilization efficiency and pesticide bioavailability. This is a new idea for achieving environmentally intelligent pesticide delivery.

## 1. Introduction

Growing crops is an important part of the agriculture industry [1,2]. Hunger currently affects 25% of the global population [3]. Insect pests decrease crop yields dramatically, either directly or by spreading diseases [4,5,6]. It has been estimated that pests decrease agricultural crop yields by ~35%. Increases in the population and decreases in the area available for arable crops mean that it is important to increase crop yields. Pesticides are important for improving crop yields [7]. However, for most pesticides, particularly insecticides, <0.1% of the pesticide reaches and acts upon the target [8]. Marked crop losses therefore still occur even when pesticides are used. Pesticides applied to agricultural land can be lost in runoff and through leaching [9], may bioaccumulate, and may enter the groundwater [10]. Pesticides therefore pose risks to ecosystems. Humans and other animals that are directly exposed to pesticides suffer health risks [11,12,13].

The fall armyworm (*Spodoptera frugiperda*) is a moth in the genus *Eustachia* in the family Noctuidae [14]. *Spodoptera frugiperda* is native to the American tropics but has a strong ability to migrate. *Spodoptera frugiperda* migrate quickly to the eastern United States and Southern Canada when the temperature increases each year. Several major insect infestations have occurred in the United States. *Spodoptera frugiperda* has spread to Africa and Asia since 2016. *Spodoptera frugiperda* has been found in 18 Chinese provinces and in Taiwan [15,16]. *Spodoptera frugiperda* is one of the most important agricultural pests around the world, harming crops in almost 100 countries and severely damaging maize and other crops. Currently, pests are mainly prevented and controlled by spraying crops with pesticides [17,18]. Recommended pesticide doses can generally effectively control pests, but doses much higher than the legal limits can control pests still more effectively, meaning that pesticides are sometimes abused [19,20].

Lambda cyhalothrin (LC) and chlorantraniliprole (Chl) are pesticides that have been recommended for controlling *Spodoptera frugiperda* in previous publications [21]. LC can inhibit conduction in insect nerve axons and is therefore toxic to insects. Chl can bind to the nicotine receptors in muscle cells in pests, which causes abnormal opening of the receptor channels and the unrestricted release of calcium ions from the calcium pool into the cytoplasm [22,23]. A wider insecticidal spectrum, faster action, and more lasting effects can be achieved using mixed formulations compared to single pesticides. A mixed pesticide formulation typically contains two pesticides dissolved in a solvent and large amounts of surfactants and other additives. Such formulations can give instant and effective pest control but cannot release pesticides slowly and in a controlled manner. Direct exposure of biota to large numbers of pesticides and additives is likely to lead to the biota being harmed [24]. Chl and LC formulations are currently typically physically mixed before being used to control *Spodoptera frugiperda*.

Nanotechnology has been used effectively in many fields and could be used in innovative ways in agriculture [25]. Applying nanoscale devices and materials can allow chemicals to be delivered in controlled and targeted ways in smart agricultural delivery systems [26]. Nanoscale microcapsules containing pesticides maintain their integrity for a long time in the complex external environment, and the active pesticide ingredients can be released slowly and in a controlled manner through dialysis [27]. The external barriers in microcapsules can allow various pesticides to be contained, meaning that previously impractical pesticide mixtures can be used. In this study, double-loaded nano-microcapsules containing Chl and LC were fabricated using a method involving the double emulsification of a solid in oil in water (S/O/W) encapsulation technique and solvent evaporation [28]. Nanomaterial pesticide delivery systems have been found to have great potential for improving pesticide efficacy. It was important that the nano-microcapsules used in this study had two compartments (the core and shell) in which Chl and LC, respectively, were localized [29]. The Chl and LC contents of the nano-microcapsules were able to be changed to meet the demands of controlling *Spodoptera frugiperda*. Synergistic effects between Chl and LC in the nano-microcapsules on controlling *Spodoptera frugiperda* were investigated. The main aim of the study was to improve our understanding of the synergistic effects between Chl and LC when co-delivered in nano-microcapsules [30,31].

## 2. Materials and Methods

### 2.1. Materials and Instruments

Polylactide (PLA; molecular weight ~100 kDa) and polyvinyl alcohol (almost 90% hydrolyzed, molecular weight 30–70 kDa) were kindly provided by Nature Works Co. (Minnetonka, MN, USA) and Sigma-Aldrich Shanghai Trading Co. (Shanghai, China), respectively. Technical LC, Chl, LC water-dispersible granules (LC-WDGs), and Chl water-dispersible granules (Chl-WDGs) were purchased from Hubei Jianyuan Chemical Co. (Wuhan, China). Chl and LC standards were purchased from J&K Chemical Technology. Bovine serum albumin and dialysis bags (with cutoffs of 8–14 kDa) were supplied by Solarbio Science Technology Co. (Beijing, China). Dichloromethane and tetrahydrofuran (AR-grade) were supplied by Macklin Biochemical Technology Co. (Beijing, China). Methanol (HPLC-grade) was supplied by Thermo Fisher Scientific Co. (Shanghai, China). The deionized water (18.2 mW cm, total organic compound concentration ≤ 4 µg/L) used in the procedures was prepared using a Milli-Q A10 system (Merck, Darmstadt, Germany).

### 2.2. Preparation of Double-Loaded Nano-Microcapsules

**Scheme 1:**
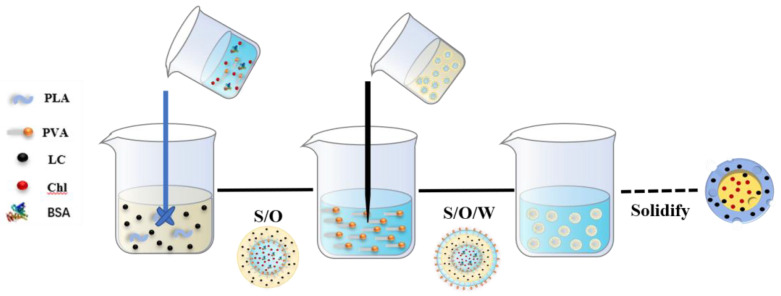
Preparation of the double-loaded nano-microcapsules containing chlorantraniliprole (Chl) and lambda cyhalothrin (LC) (PLA = polylactide, PVA = polyvinyl alcohol, BSA = bovine serum albumin, O = oil, S = solid, W = water, S/O = solid in oil, W/O = water in oil, S/O/W = solid in oil in water).

The double emulsification S/O/W and solvent evaporation method was used to prepare the double-loaded nano-microcapsules. The method has been described previously but was used with some modifications because of the poor solubility of Chl in water [32,33]. Briefly, 0.6 g of PLA and 40 mg of LC was completely dissolved in 20 mL of dichloromethane. This was the oil phase. Then, 0.5 g of bovine serum albumin and 0.05 g of polyvinyl alcohol were dissolved in 5 mL of deionized water, and 40 mg of Chl was dispersed in the solution. This was the solid phase. The solid phase was poured into the oil phase, and the mixture was oscillated at 10,000 rpm for 1 min to give a solid in oil (S/O) emulsion. The S/O emulsion was added to 120 mL of water containing 0.2% polyvinyl alcohol, and the mixture was ultrasonicated using a JY90-IIN instrument (Ningbo, China) for 3 min at 30–60% of the maximum power. The double emulsion was then mixed at 500 rpm for 10 h until the organic solvent had evaporated; then, the milky white mixture was centrifuged at 10,000 rpm for 10 min at 10 °C. The supernatant was discarded and the same amount of deionized water was added. The final centrifuging procedure was repeated three times, with deionized water added each time, to give the double-loaded nano-microcapsules, as shown in Scheme 1. The nano-microcapsules were then lyophilized for 2 d to give the double-loaded nano-microcapsules as a white powder.

### 2.3. Characterization of the Nano-Microcapsules

The nano-microcapsules were dispersed in deionized water and diluted to a concentration of 0.5%. The morphological features of the nano-microcapsules were investigated by scanning electron microscopy (SEM), using an SU8010 instrument (Hitachi, Tokyo, Japan) with an acceleration voltage of 5 kV, and by transmission electron microscopy (TEM), using an HT7700 instrument (Hitachi, Tokyo, Japan) with a voltage of 80 kV. The nano-microcapsule particle sizes and polydispersity index (PDI) were determined using a dynamic laser scattering (DLS) instrument (Zetasizer Nano ZS90, Malvern Panalytical, Malvern, UK). Each sample was analyzed in triplicate to ensure that the data were reliable. The nano-microcapsules were analyzed by Fourier-transform infrared spectroscopy using the KBr method. The LC and Chl concentrations were determined by high-performance liquid chromatography (HPLC) using an Agilent 1260 series HPLC instrument (Agilent Technologies, Santa Clara, CA, USA). Environmental SEM images of the nano-microcapsules were acquired using an FEI Quanta FEG 250 SEM instrument (Thermo Fisher Scientific, Waltham, MA, USA).

### 2.4. Drug Loading Content and Encapsulation Efficiency

The LC and Chl drug loading contents were determined using an Agilent 1260 series HPLC instrument fitted with a Zorbax Carbon hydrate analysis column (150 mm long, 4.6 mm i.d., 5 µm particle size; Agilent Technologies). The HPLC settings are shown in Table 1. A specified amount of the freeze-dried nano-microcapsules was dissolved in tetrahydrofuran; then, the LC and Chl concentrations were determined by HPLC. The encapsulation efficiency was determined by determining the LC and Chl concentrations in the nano-microcapsules and supernatant after centrifugation using the same method. The equations used to calculate the drug loading content and encapsulation efficiency are shown below.

(1) $$\mathrm{Drug}\;\mathrm{loading}\;\mathrm{content}\;\%=\frac{\mathrm{weight}\;\mathrm{of}\;\mathrm{LC}/\mathrm{Chl}\;\mathrm{in}\;\mathrm{lyophilized}\;\mathrm{microcapsules}}{\mathrm{weight}\;\mathrm{of}\;\mathrm{lyophilized}\;\mathrm{microcapsules}}\times \;100\;\%$$

(2) $$\mathrm{Encapsulation}\;\mathrm{effiency}\;\%=\frac{\mathrm{weight}\;\mathrm{of}\;\mathrm{LC}/\mathrm{Chl}\;\mathrm{in}\;\mathrm{lyophilized}\;\mathrm{microcapsules}}{\mathrm{weight}\;\mathrm{of}\;\mathrm{input}\;\mathrm{LC}/\mathrm{Chl}}\times \;100\;\%$$

### 2.5. Pesticide Release from the Nano-Microcapsules

The dialysis bag method was used to investigate the release of Chl and LC in vitro [34,35]. A specified amount of the nano-microcapsules was dispersed in 10 mL of the release medium (a 9:1 *v*/*v* methanol: water mixture); then, the suspension was transferred into a dialysis bag. The two ends of the dialysis bag were closed with clamps; then, the bag was placed into a 150 mL glass bottle containing 95 mL of release medium. At a specified time, a 2 mL aliquot of the dialysate was removed and 2 mL of release medium was added to maintain the volume in the bottle. The Chl and LC concentrations in the dialysate were determined by HPLC following the method described above.

### 2.6. Stability of the Nano-Microcapsules Stored at Different Temperatures

The stability of a pesticide during storage at various temperatures is a very important criterion. Diluted nano-microcapsule suspensions were stored at 0, 25, and 54 °C for 14 d in incubators. The hydrodynamic particle sizes and polydispersity indices (PDIs) of the samples were determined every 2 d during the storage period using the DLS instrument described above.

### 2.7. Indoor Control Studies Using Spodoptera Frugiperda

The spraying method was used to determine how effective the double-loaded nano-microcapsules, commercial formulations, and physical mixtures (LC-WDGs, Chl-WDGs, and a mixture of LC-WDGs and Chl-WDGs) were. The pesticide concentrations that were used were 12, 6, 3, 1.5, 0.75, and 0.375 mg/L, and treatment without pesticide was used as a blank control. Artificial feed was sliced and placed in 6 cm diameter plastic petri dishes. Third-instar larvae of *Spodoptera frugiperda* were gently placed on the artificial feed with a brush, with 15 larvae per dish. Each dish was then placed under a tower and accurately sprayed with 2 mL of the test suspension at a pressure of 68.9 kPa. After being sprayed, each dish was left for 30 s and then placed in an incubator at 25 ± 2 °C. Each treatment was performed in triplicate. After 48 h, the larvae were examined using an anatomical microscope and the number of dead larvae was recorded. The concentration that killed 50% of the larvae (LC_50_) and the toxicity regression equation were determined.

### 2.8. Wettability of the Foliage Treated with the Nano-Microcapsules

A contact angle measuring instrument (JC2000D2 M; Zhongshan Digital Technology Apparatus, Shanghai, China) was used to determine the contact angles for the pesticide formulations (technical LC, technical Chl, LC-WDGs, Chl-WDGs, and the nano-microcapsule suspension) on cucumber and cabbage leaves. The effective concentration of each was adjusted to 0.04% to mimic the concentration of commercial water-dispersible granules recommended for spraying outdoors on fields. Cucumber and cabbage leaves grown in an indoor incubator were washed with deionized water and allowed to dry naturally. Leaves of appropriate sizes were then fixed to glass slides using double-sided adhesive tape, avoiding damaging the leaf structure. A slide was placed on the loading platform of the contact angle measuring instrument and the height was adjusted so that the leaf was in the CCD camera collection area. A 7 µL aliquot of a sample was added using a microsyringe. An image of the point of contact between the liquid and the leaf was acquired after the droplet had been in contact with the leaf for 30 s. A five-point fitting method was used to analyze the contact angles.

### 2.9. Statistical Analysis

Data analysis was performed using SPSS 20 software (IBM, Armonk, NY, USA). The data were analyzed by performing one-way analyses of variance and Duncan’s multiple range tests, using *p* < 0.05 to indicate statistical significance.

## 3. Results and Discussion

### 3.1. Double-Loaded Nano-Microcapsule Preparation

The solubilities of Chl and LC in water and organic solvents are different. The S/O/W pesticide packaging technique was used to simultaneously encapsulate Chl and LC to improve the compatibility of these ‘mismatched’ pesticides. LC is soluble in organic solvents but Chl is poorly soluble in organic solvents or water. Chl was directly dispersed in deionized water to prepare a solid suspension of nanosized particles (the solid phase). LC was dissolved in dichloromethane, and the solution was used as the oil phase. Bovine serum albumin was added to act as a penetrant to give an S/O emulsion (similar to a water in oil emulsion produced through high-speed shear). The S/O emulsion was then added to the water phase, and the mixture was ultrasonicated to give a double emulsification (S/O/W). The ratio of Chl and LC can be changed from 1:3 to 3:1 with this method (Table 1). The Chl and LC double-loaded microcapsule suspension was successfully produced using this simple double emulsification curing method. The 1:1 ratio of Chl and LC was adopted to fabricate double-loaded nano-microcapsules for the next study.

**Figure 1:**
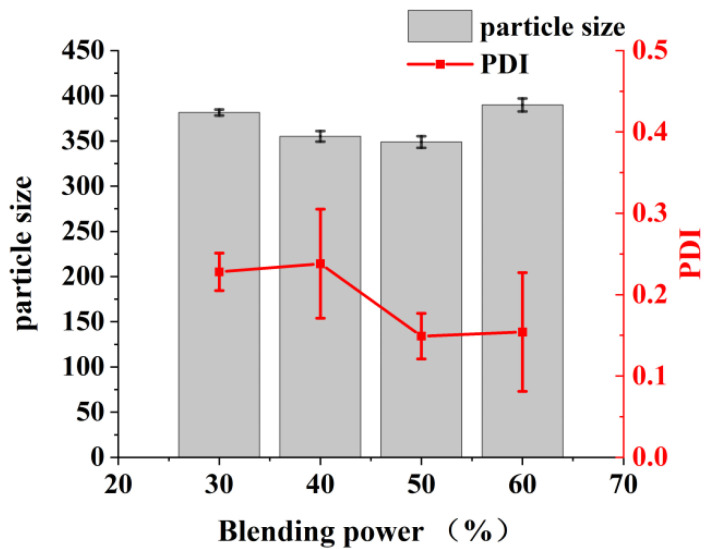
Hydrodynamic particle sizes and polydispersity indices (PDIs) determined by dynamic laser scattering after different emulsification powers had been used.

The mean hydrodynamic particle size and PDI for the nano-microcapsule suspension were determined using a DLS system and are shown in Figure 1. The minimum hydrodynamic particle size at an emulsifying power of 50% was 348.9 ± 6.4 nm. Increasing the emulsifying power caused the nano-microcapsule particle size to gradually decrease. However, increasing the power to a certain point caused the particle size to increase. This would have been because strong tearing of the material during phacoemulsification can quickly and dramatically decrease the droplet size. However, using too high a power would give a high surface energy that would cause the dispersed small particles to gather together again, which would not be conducive to the stability of the solution. The PDIs of the four samples with different particle sizes were all <0.25, indicating that the molecular weight distribution was narrow and the particle size distribution was relatively uniform [36,37].

### 3.2. Nano-Microcapsules’ Morphologies and Characteristics

**Figure 2:**
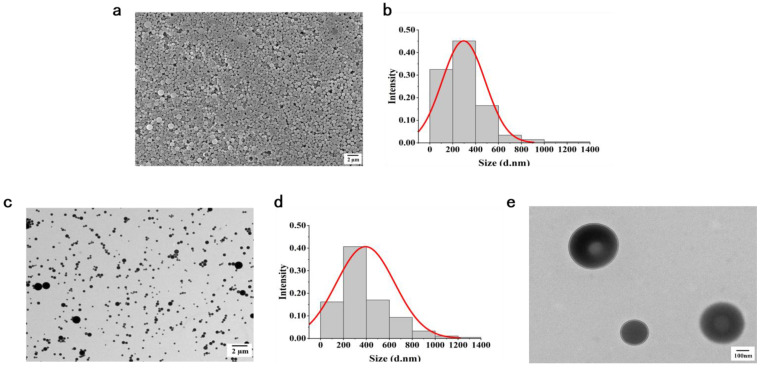
Electron microscopy images of the nano-microcapsules. (**a**) Scanning electron microscopy image, (**b**) size distribution based on the scanning electron microscopy data for 100 particles, (**c**) transmission electron microscopy image, (**d**) size distribution based on the transmission electron microscopy data for 100 particles, (**e**) high-magnification TEM image. The scale bars in the images indicate 1.0 µm.

The three- and two-dimensional structures of the nano-microcapsules were investigated by SEM and TEM. As shown in Figure 2a,b, the TEM images indicated that the particles were spherical with a clear core–shell structure, and the SEM images indicated that some of the particles contained small holes (Figure 2e). The mean particle diameter was <400 nm (Figure 2b,d), which agreed with the DLS results.

**Figure 3:**
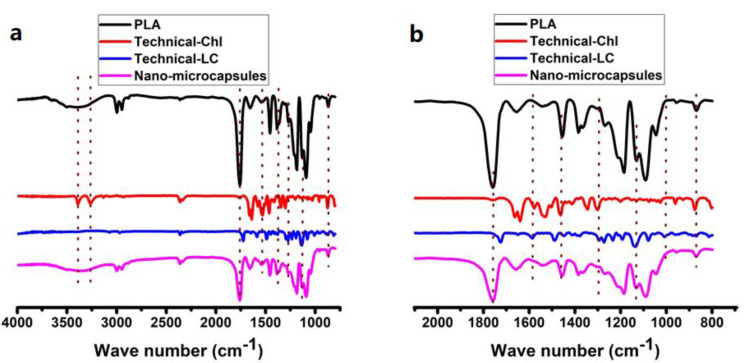
(**a**) Fourier-transform infrared spectra of polylactide (PLA), chlorantraniliprole (Chl), lambda cyhalothrin (LC), and the double-loaded nano-microcapsules, and (**b**) magnified regions of the spectra.

Fourier-transform infrared spectroscopy can be used to identify a substance from the characteristic absorption peaks of specific groups in the substance. Fourier-transform infrared spectroscopy was performed to investigate Chl and LC encapsulation [38]. The nano-microcapsule Fourier-transform infrared spectrum shown in Figure 3 contained strong peaks at 1091, 1184, and 1755 cm^−1^ corresponding to symmetric stretching vibration and asymmetric stretching vibration of C–O–C=O, and stretching vibration of C=O, that were attributed to PLA. Peaks at 3386 and 1635 cm^−1^ corresponding to amide N–H stretching vibration and amide C=O stretching vibration were attributed to Chl, and a peak at 1130 and 1720 cm^−1^ corresponding to symmetric stretching vibration of C–O–C and stretching vibration of C=O was attributed to LC [39]. Other peaks characteristic of Chl and LC were not found, possibly because of strong absorbance by the PLA wall material, which would have masked absorbance by the active ingredients. These results indicated that Chl and LC were successfully simultaneously encapsulated in the nano-microcapsules [40].

### 3.3. Loading Content and Encapsulation Efficiency

**Figure 4:**
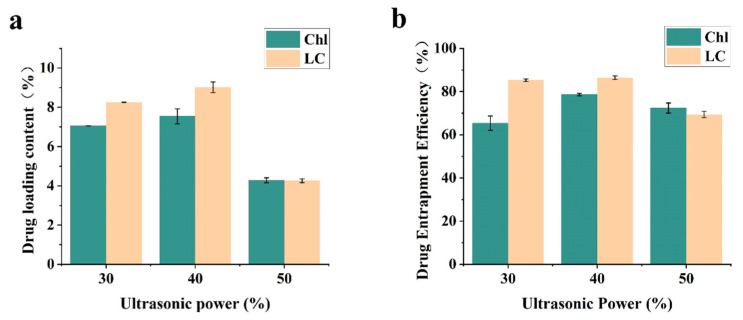
(**a**) Drug loading contents and (**b**) encapsulation efficiencies for chlorantraniliprole (Chl) and lambda cyhalothrin (LC) at different ultrasonic emulsification powers.

**Figure 5:**
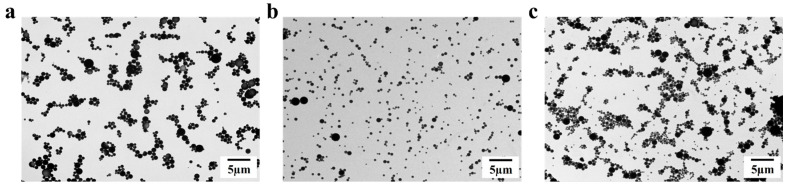
Transmission electron microscopy images of nano-microcapsules produced using emulsifying powers of (**a**) 30%, (**b**) 40%, and (**c**) 50%. The scale bar indicates 5 µm.

The encapsulation efficiency and drug loading content are important factors when evaluating fabrication techniques for nano drug loading systems. Higher loadings and encapsulation efficiencies will minimize the waste of active ingredients. Several nano-microcapsules were constructed using different ultrasonic emulsification powers to determine which emulsification power maximized the pesticide utilization efficiency. As shown in Figure 4, the Chl and LC encapsulation efficiencies were >65%. The encapsulation efficiency was higher for LC than Chl when the ultrasonic emulsification power was 40%. The LC and Chl encapsulation efficiencies and drug loading contents were highest when the ultrasonic emulsification power was 40%. It could be expected that too low an ultrasonic emulsification power would give a poor encapsulation efficiency but that too high an ultrasonic emulsification power would rupture the microcapsules (Figure 5), leading to pesticides leaking from the microcapsules. The ultrasonic emulsification procedure was therefore performed using an ultrasonic emulsification power of 40%, which gave Chl and LC encapsulation efficiencies of 78% and 86%, respectively, and drug loading contents of 7.4% and 9.2%, respectively.

### 3.4. Pesticide Release Behavior

**Figure 6:**
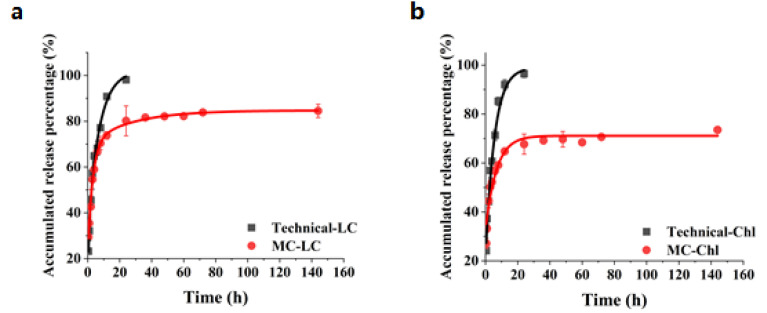
Cumulative release curves of technical and nano microcapsules. (**a**) Lambda Cyhalothrin (LC) and (**b**) Chlorantraniliprole (Chl).

The release of Chl and LC from the double-loaded pesticide nano-microcapsules in a mixture of methanol and water at room temperature was explored and compared with the release of technical Chl and technical LC. The double-loaded pesticide nano-microcapsules slowly and simultaneously released Chl and LC [41]. As shown in Figure 6, technical Chl and technical LC were released rapidly and reached maximum release rates within 24 h, but the nano-microcapsules released Chl and LC more slowly, indicating that encapsulation slowed pesticide release. The cumulative Chl and LC release profiles were quite similar. The cumulative percentage of LC released at 40 h was >80%, and the cumulative percentage of Chl released at 40 h was <70%. Chl was released more slowly than LC from the double-loaded pesticide nano-microcapsules because LC was within the wall material and Chl was within the core. The cumulative release results indicated that the nano-microcapsules released Chl and LC in a sustained manner.

### 3.5. Stability of the Nano-Microcapsules Stored at Different Temperatures

**Figure 7:**
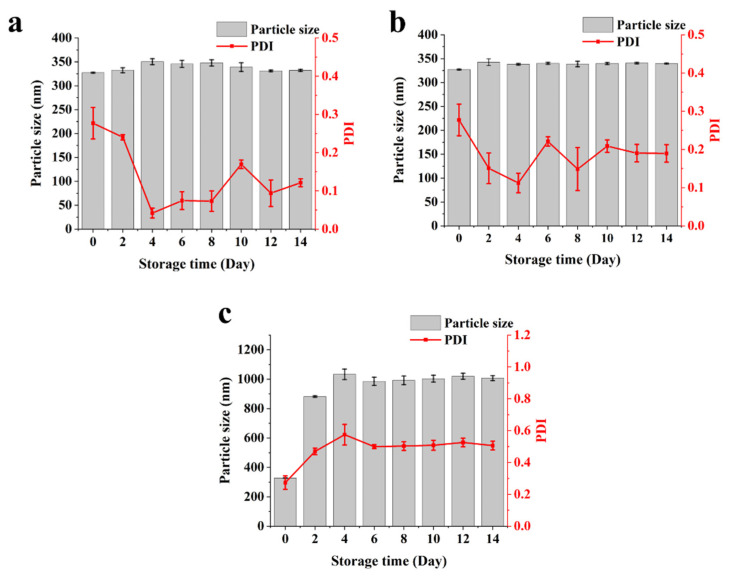
Particle sizes and polydispersity indices (PDIs) of double-loaded nano-microcapsules stored at (**a**) 0 °C, (**b**) 25 °C, and (**c**) 54 °C.

**Figure 8:**
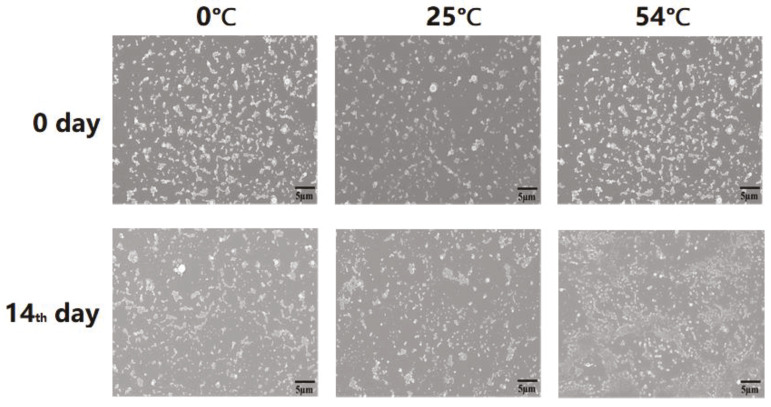
Scanning electron microscopy image changes of nano-microcapsules stored at 0, 25, and 54 °C for 14 days.

The thermal stability of a pesticide formulation is important when the formulation is transported and stored. Changes in the mean hydrodynamic sizes and PDIs of the nano-microcapsules stored at 0, 25, and 54 °C for 14 d were determined by DLS to assess the thermal stability of the nano-microcapsules. As shown in Figure 7a, the mean hydrodynamic size of the nano-microcapsules in suspension increased by around 3.9% (from 327 to 340 nm) during storage at 25 °C for 14 d. As shown in Figure 7b, the mean hydrodynamic size of the nano-microcapsules in suspension increased by only 1.5% during storage at 0 °C for 14 d. The PDIs for nano-microcapsules in suspension stored at 4 and 25 °C for 14 d were <0.2, indicating that the nano-microcapsules had narrow distributions. The mean hydrodynamic size increased by >200% when the nano-microcapsules in suspension were stored at 54 °C for 14 d (Figure 7c). The PDI and mean hydrodynamic size both increased markedly when the nano-microcapsules were stored at 54 °C, presumably because this is very close to the PLA glass transition temperature. The PLA shell would have become soft at 54 °C, causing the nano-microcapsules to stick together (Figure 8). The Chl and LC contents of the nano-microcapsules stored at different temperatures were determined by HPLC and were found to have remained almost unchanged. These results indicated that the nano-microcapsules were stable at a low temperature (0 °C) and at room temperature (25 °C) for a long time.

### 3.6. Wettability Evaluation

**Figure 9:**
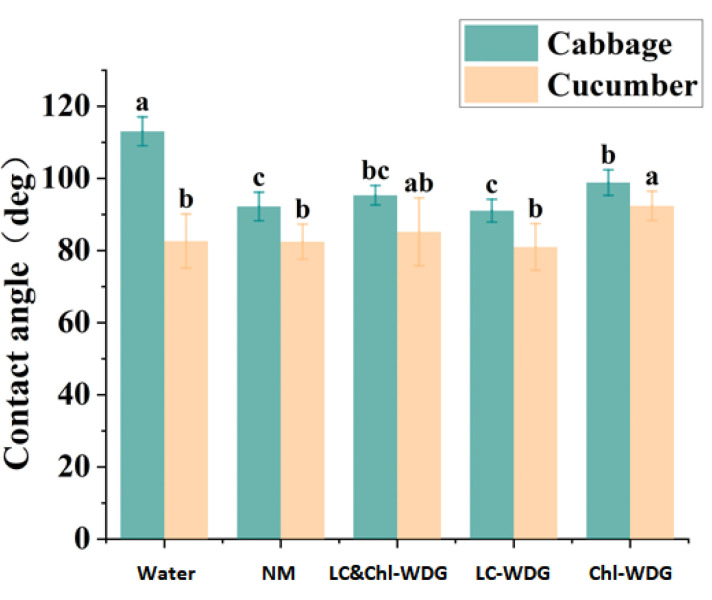
Contact angles for droplets of water, the nano-microcapsules containing lambda cyhalothrin and chlorantraniliprole (NM), LC-WDGs mixed with Chl-WDGs (LC and Chl-WDG), LC-WDG, and Chl-WDG on cucumber and cabbage leaf surfaces. The different letters above columns indicate significant differences among different treatments (one-way ANOVA, Duncan test, *p* < 0.05).

The contact angle of a droplet on the surface of a crop leaf is an important parameter when determining the ability of a liquid to wet the crop leaf surface. A droplet will more readily spread along a leaf if the contact angle of the droplet is small compared to when the contact angle is large, meaning that less pesticide will be lost if the contact angle is small compared to when the contact angle is large. As shown in Figure 9, the contact angles of deionized water droplets on the cucumber and cabbage leaves were 83 ± 5° and 113 ± 4°, respectively. This suggested that the cucumber leaf surface was weakly hydrophilic and the cabbage leaf was hydrophobic (the hydrophilic–hydrophobic boundary is 90°). The contact angles of droplets of the suspensions of double-loaded nano-microcapsules, LC-WDGs, Chl-WDGs, and LC-WDGs mixed with Chl-WDGs on the cucumber leaf surfaces were 82 ± 4°, 81 ± 6°, 92 ± 4°, and 85 ± 6°, respectively. The contact angles of droplets of suspensions of the double-loaded nano-microcapsules, LC-WDGs, Chl-WDGs, and LC-WDGs mixed with Chl-WDGs on the cabbage leaf surfaces were 92 ± 4°, 91 ± 3°, 99 ± 4°, and 95 ± 3°, respectively. This indicated that the wettability of the cucumber leaf surfaces was slightly better for the formulations than deionized water, and the wettability of cabbage leaf surfaces was markedly better for the formulations than deionized water. The contact angles were slightly smaller for the nano-microcapsule suspension than for the Chl-WDGs and LC-WDGs mixed with Chl-WDGs. The contact angle was relatively small for the LC-WDGs because the surfactant concentration was much higher than for the other suspensions.

### 3.7. The Adhesion of Nano-Microcapsules on the Surfaces of Crop Leaves

**Figure 10:**
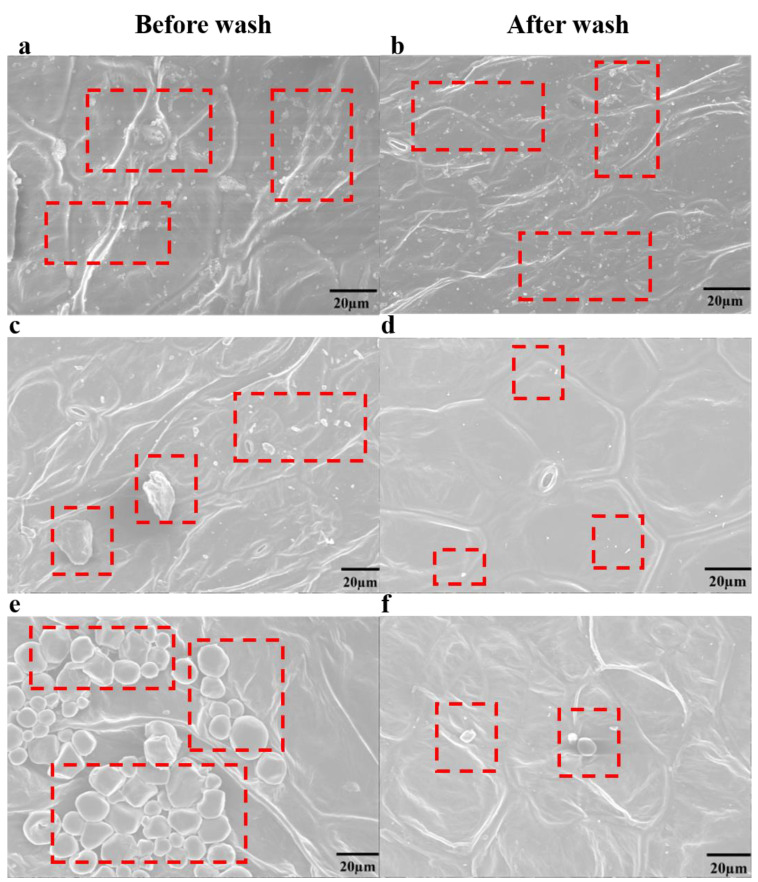
SEM images on cabbage leaves. (**a**) Image with nano-microcapsules, (**c**) image with WDG-Chl, (**e**) image with WDG-LC. (**b**,**d**,**f**) are the corresponding images after washing. The scale bar is 20 µm.

**Figure 11:**
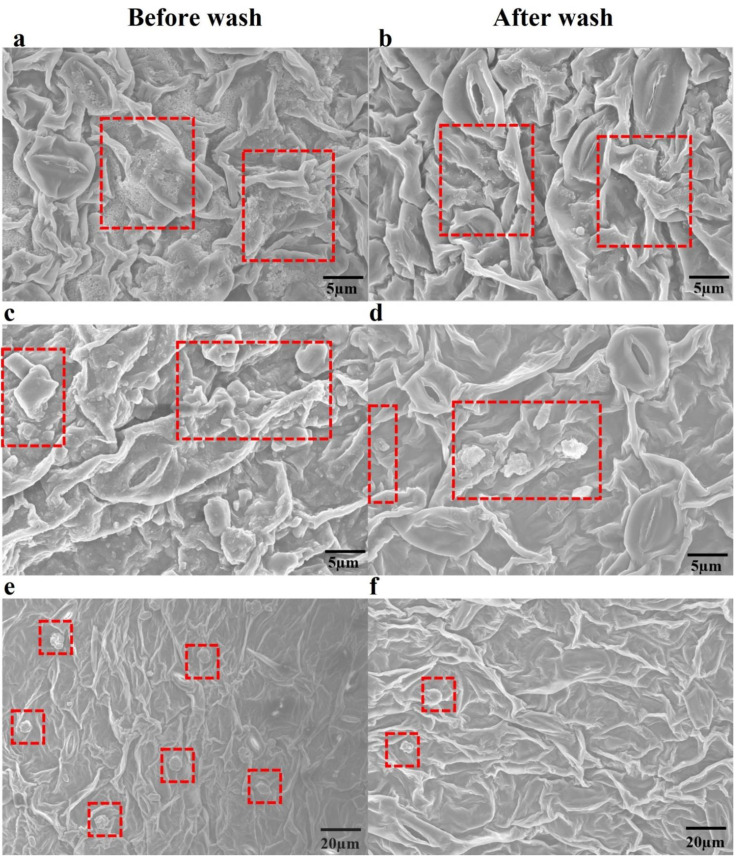
SEM images on cucumber leaves. (**a**) Image with nano-microcapsules, (**c**) image with WDG-Chl, (**e**) image with WDG-LC. (**b**,**d**,**f**) are the corresponding images after wash. The scale bar is 5 µm for (**a**–**d**) and 20 µm for (**d**,**e**).

To obtain a vivid visualization of pesticide particles on the surfaces of crop leaves, SEM was applied to characterize deposit and adhesion behaviors. As shown in Figure 10 and Figure 11, a large number of nano-microcapsules deposited everywhere on the crop surfaces, and the particle numbers of nano-microcapsules were much greater than LC-WDG and Chl-WDG. Due to the size of nano-microcapsules being far less than that of stoma, the nano-microcapsules might be inhaled via a “stoma pathway”, and subsequently be transported to inner parts of leaves. After washing with deionized water simulating rain wash, plenty of nano-microcapsules remaining were discerned, and most of the large particles of LC-WDGs and Chl-WDGs were washed away from the leaf surface, with very few small particles remaining. These results suggested that the nano-microcapsules showed better adhesion to crop leaves.

### 3.8. Bio-Control to Spodoptera Frugiperda

The spraying method was used to evaluate the efficiency with which *Spodoptera frugiperda* were controlled by the nano-microcapsules, LC-WDGs, Chl-WDGs, and the mixture of LC-WDGs and Chl-WDGs. The results are shown in Table 2. *Spodoptera frugiperda* were controlled less effectively by the LC-WDGs than the Chl-WDGs because *Spodoptera frugiperda* are somewhat resistant to LC. Little synergism occurred (relative to applying LC-WDG and Chl-WDG separately) when the mixture of LC-WDGs and Chl-WDGs was used to control *Spodoptera frugiperda*, and the LC_50_ was 1.05 mg/L. *Spodoptera frugiperda* were controlled markedly more effectively by the nano-microcapsules than the LC-WDGs and Chl-WDGs, the LC_50_ for the nano-microcapsules being 0.39 mg/L, which was much lower than the LC_50_ for the mixture of LC-WDGs and Chl-WDGs. This clearly indicated that the nano-microcapsules containing Chl and LC in the core–shell structure caused a remarkable synergistic enhancement in *Spodoptera frugiperda* control. Using the nano-microcapsules would therefore improve the efficiency with which *Spodoptera frugiperda* are controlled and also increase the pesticide utilization efficiency.

## 4. Conclusions

We developed a fast, simple, and controllable method for preparing double-loaded nano-microcapsules to control *Spodoptera frugiperda*. An improved S/O/W encapsulation technique was used to encapsulate poorly water-soluble Chl in the nanosized microcapsule core and LC in the wall. This increased the drug content without using a large amount of surfactant (much less than is used in commercial pesticide formulations). The nano-microcapsules released both pesticides slowly and adhered well to crop leaf surfaces compared with commercial formulations. The *Spodoptera frugiperda* control efficiency was markedly higher for LC and Chl in the nano-microcapsules than for LC-WDGs, Chl-WDGs, and a mixture of LC-WDGs and Chl-WDGs because of the synergistic enhancement of the effects of Chl and LC. The process used to prepare these nano-microcapsules is suitable for the practical production of other nano-microcapsules, and using such nano-microcapsules would be less harmful to the environment than using other pesticide formulations. The nano-microcapsules can be produced to give a good degree of insecticide efficiency and to slowly release multiple pesticides in practice. Nano-microcapsules could therefore improve the utilization efficiencies of multiple pesticides, which would offer great advantages for future applications.

## Figures and Tables

**Table 1:** The hydrodynamic particle size and PDI with different ratios of Chl to LC, measured by DLS.

Ratio of Chl to LC	Particle Size (nm)	PDI
1:1	313.9 ± 1.1	0.215 ± 0.02
1:3	273.4 ± 1.2	0.166 ± 0.04
3:1	273.0 ± 3.7	0.169 ± 0.08

**Table 2:** Toxicities of the nano-microcapsules and commercially available water-dispersible granules.

Formulations	Regressive Equation	R-Square	LC_50_ (mg/L)
LC-WDG	y = 0.56 + 3.55x	0.93	42.2
Chl-WDG	y = 4.96 + 1.79x	0.91	1.52
LC and Chl-WDG	y = 1.38 + 1.65x	0.95	1.05
Nano-microcapsules	y = 5.37 + 1.38x	0.92	0.39

## Data Availability

The data presented in this study are available on request from the corresponding author.
